# Three resected cases of esophageal carcinoma considered as being secondary solid tumors after bone marrow transplantation

**DOI:** 10.1186/s40792-021-01157-z

**Published:** 2021-03-20

**Authors:** Yamato Ninomiya, Soji Ozawa, Kazuo Koyanagi, Miho Yamamoto, Tadashi Higuchi, Kentaro Yatabe, Kohei Tajima

**Affiliations:** Department of Gastroenterological Surgery, Tokai University School of Medicine, 143 Shimokasuya, Isehara, Kanagawa 259-1193 Japan

**Keywords:** Esophageal cancer, Bone marrow transplantation, Secondary solid tumors, Chronic graft-versus-host disease

## Abstract

**Background:**

Bone marrow transplantation is now an established treatment for some hematopoietic disorders and hematopoietic malignancies, and secondary solid tumors that develop after bone marrow transplantation have begun to attract attention.

**Case presentation:**

Herein, we report 3 cases of esophageal carcinoma that developed after bone marrow transplantation. Case 1: 40-year-old female received cyclophosphamide and total body irradiation at 12 Gy for acute myeloid leukemia, followed by related bone marrow transplantation. She developed chronic graft-versus-host disease manifesting as pulmonary complications and was administered cyclosporine. Nine years after the transplantation, she was diagnosed as having esophageal carcinoma Stage II and underwent radical surgery. She died of the primary disease 17 months after the surgery. Case 2: A 45-year-old male patient received cyclophosphamide, VP-16 and total body irradiation at 13.2 Gy for acute lymphocytic leukemia, followed by related bone marrow transplantation. He developed chronic graft-versus-host disease manifesting as liver dysfunction. Fifteen years after the transplantation, he was diagnosed as having esophageal carcinoma Stage II and underwent radical surgery. Seven months after the surgery, he died of the primary disease. Case 3: A 30-year-old female patient received cyclophosphamide and total body irradiation at 3 Gy for Fanconi anemia, followed by unrelated bone marrow transplantation. She developed chronic graft-versus-host disease manifesting as a rash and was administered tacrolimus and methotrexate. Fifteen years after the transplantation, she was diagnosed as having esophageal carcinoma Stage III and underwent radical surgery. She died of sepsis 7 months after the surgery.

**Conclusion:**

The esophageal carcinomas developing after bone marrow transplantation had the characteristics of secondary solid tumors in all 3 patients, such as early onset, after total body irradiation, association with chronic graft-versus-host disease, and history of use of immunosuppressive drugs. Patients undergoing bone marrow transplantation require long-term follow-up after the transplantation, considering the possible development of secondary solid tumors, and in regard to secondary solid tumors developing in the gastrointestinal tract, it must be borne in mind that the risk of esophageal carcinoma is particularly high.

## Background

Bone marrow transplantation (BMT) is used to treat many hematopoietic disorders and hematopoietic malignancies, and with the increasing number of cases and improved treatment outcomes, late complications occurring in long-surviving patients have begun to attract attention. Secondary solid tumors (SSTs) that develop after BMT are among the reported late complications, and recently there have been both isolated case reports and reports of large-scale studies of SSTs; however, few cases of esophageal carcinoma as SSTs have been reported until now [1–4]. Herein, we report 3 resected cases of esophageal carcinoma, considered as SSTs, after BMT.

## Cases presentation

### Case 1: a 40-year-old woman

**Fig. 1:**
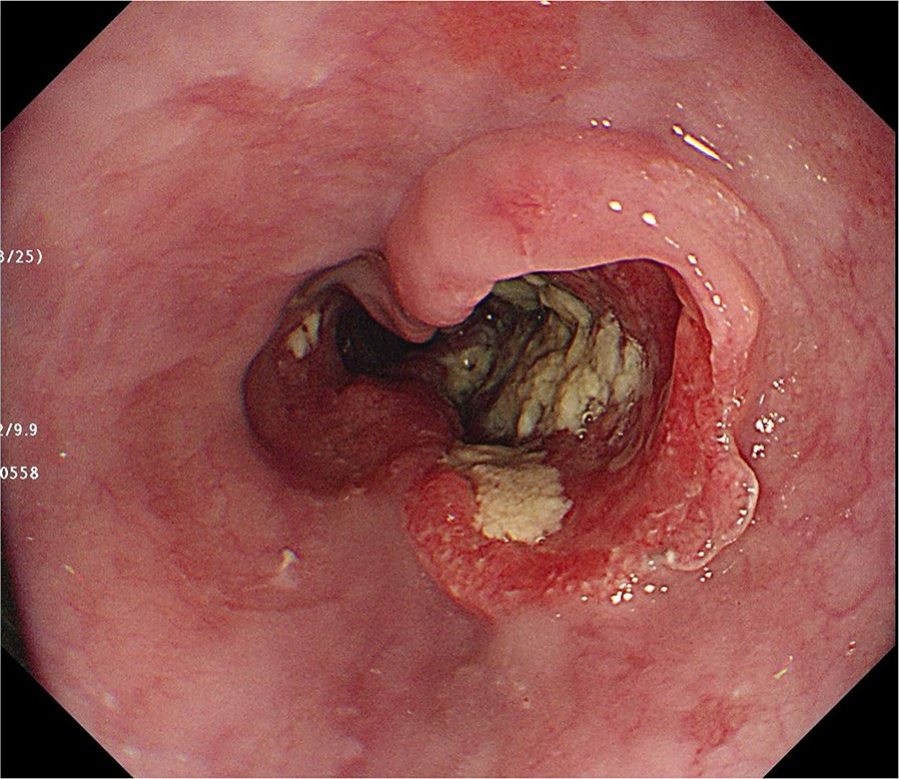
Endoscopic findings in Case 1. A type 2 lesion was found in the lower thoracic esophagus

**Fig. 2:**
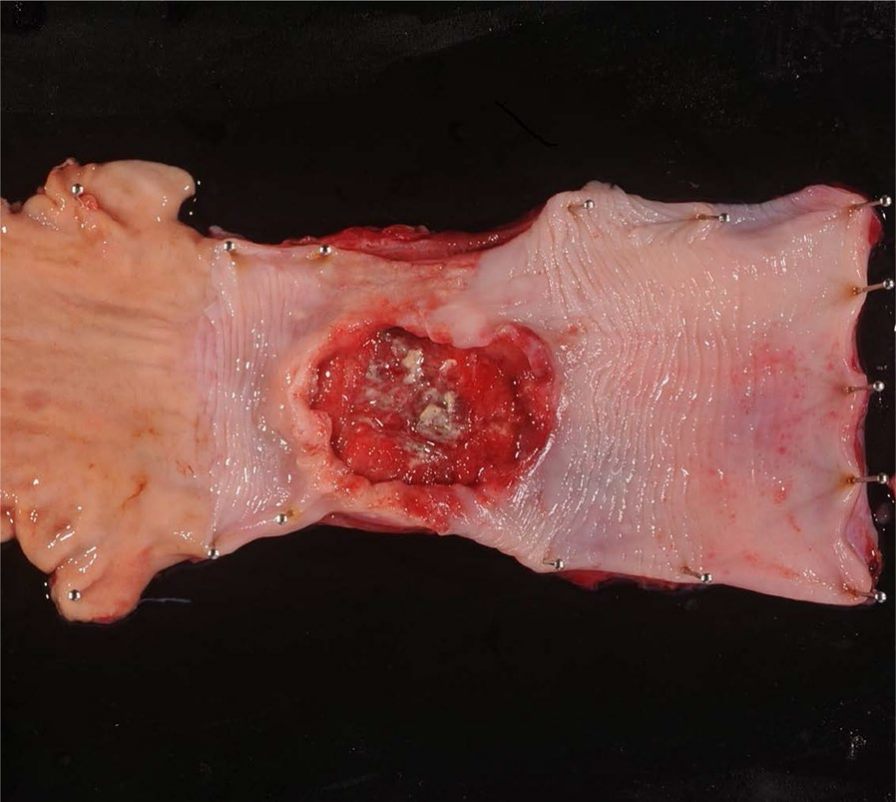
Findings of the resected specimen in Case 1

She had undergone related BMT from her older human leukocyte antigen (HLA)-matched sister at the age of 31 years for acute myeloid leukemia (M2), which was diagnosed when she was 30 years old. She received cyclophosphamide and total body irradiation at 12 Gy as pretreatment. Approximately 5 months after the BMT, she developed post-transplant chronic graft-versus-host disease (GVHD) manifesting as pulmonary complications, and received treatment with cyclosporine. Subsequently, she had no recurrence and was followed up on an outpatient basis. Nine years after the BMT, she became aware of difficulty in swallowing after meals and presented to our department. She had never undergone upper gastrointestinal endoscopy and had a 20-year history of habitual drinking of 2000 ml of beer/day and smoking 20 cigarettes/day. Detailed examination led to the diagnosis of esophageal carcinoma (Lt, Type 2, T3N0M0 Stage II) (Fig. 1). We thought that she was a high-risk case for neoadjuvant chemotherapy (NAC) as she had undergone BMT, and instead performed thoracoscopic thoracic esophagectomy and retrosternal gastric conduit reconstruction. She was discharged on the 20th postoperative day without any postoperative complications. The histopathological diagnosis of the resected specimen was moderately differentiated squamous cell carcinoma, Type 2, pT3N3 (8/94 metastasis positive lymph nodes) M0, pStage III, INFb, ly1, v2 (Fig. 2). She developed multiple lymph node and pulmonary metastases 12 months after the surgery and underwent a total of 4 courses of chemotherapy (CF therapy: cisplatin 80 mg/m^2^ and 5-fluorouracil 800 mg/m^2^). She died of progressive esophageal carcinoma 17 months after the surgery.

### Case 2: a 45-year-old man

**Fig. 3:**
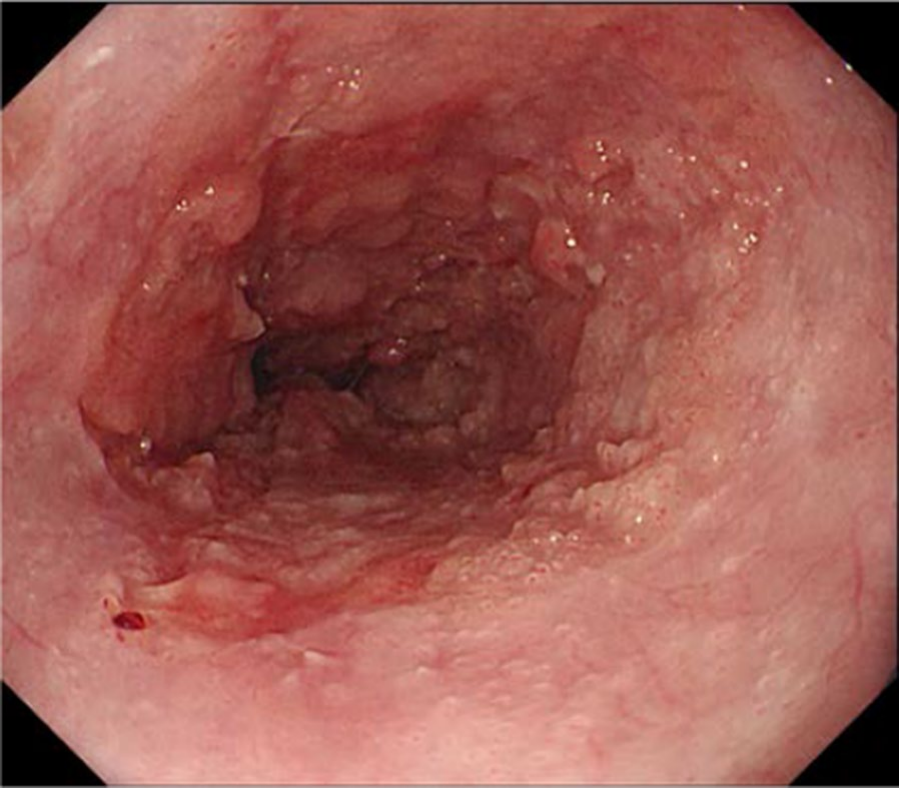
Endoscopic findings in Case 2. A type 3 lesion was found in the lower thoracic esophagus

**Fig. 4:**
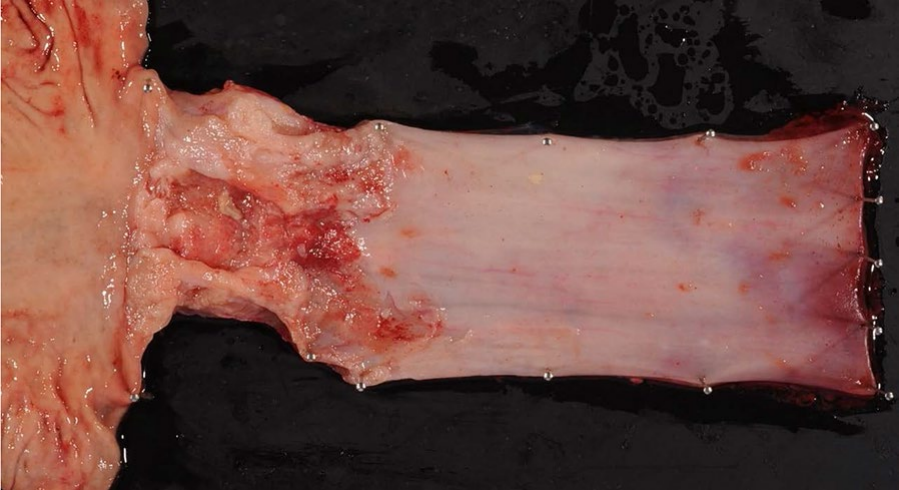
Findings of the resected specimen in Case 2

He was diagnosed as having acute lymphocytic leukemia at the age of 29 years and had undergone related BMT from his younger HLA-matched sister at the age of age 30 years. He received cyclophosphamide, VP-16 and total body irradiation at 13.2 Gy as pretreatment. After transplantation, he developed chronic GVHD manifesting as liver dysfunction. Fifteen years after the BMT, he became aware of difficulty in swallowing and presented to our department. He had never undergone upper gastrointestinal endoscopy and was not a smoker, but had a 25-year history of habitual drinking of 350 ml of beer/day. Detailed examination led to the diagnosis of esophageal carcinoma (LtAe, Type 3, T2N0M0 Stage II) (Fig. 3). And as in Case 1, he did not receive NAC, but underwent thoracoscopic thoracic esophagectomy with retrosternal gastric conduit reconstruction. He was discharged on the 19th postoperative day without any postoperative complications. The histopathological diagnosis of the resected specimen was well differentiated squamous cell carcinoma, Type 3, pT3N2 (2/56 metastasis positive lymph nodes) M0, pStage III, Infb, ly2, v1 (Fig. 4). He developed multiple pulmonary metastases 6 months after surgery and died of progressive esophageal carcinoma 7 months after surgery.

### Case 3: a 30-year-old woman

**Fig. 5:**
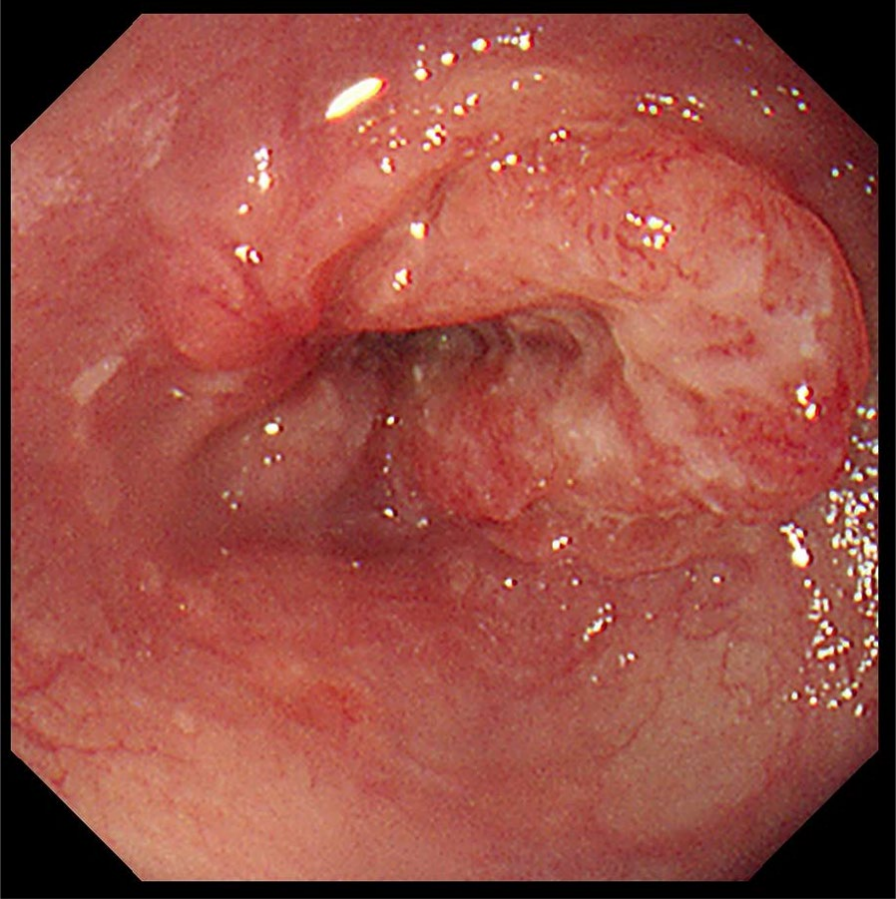
Endoscopic findings in Case 3. A type 2 lesion was found in the middle thoracic esophagus

**Fig. 6:**
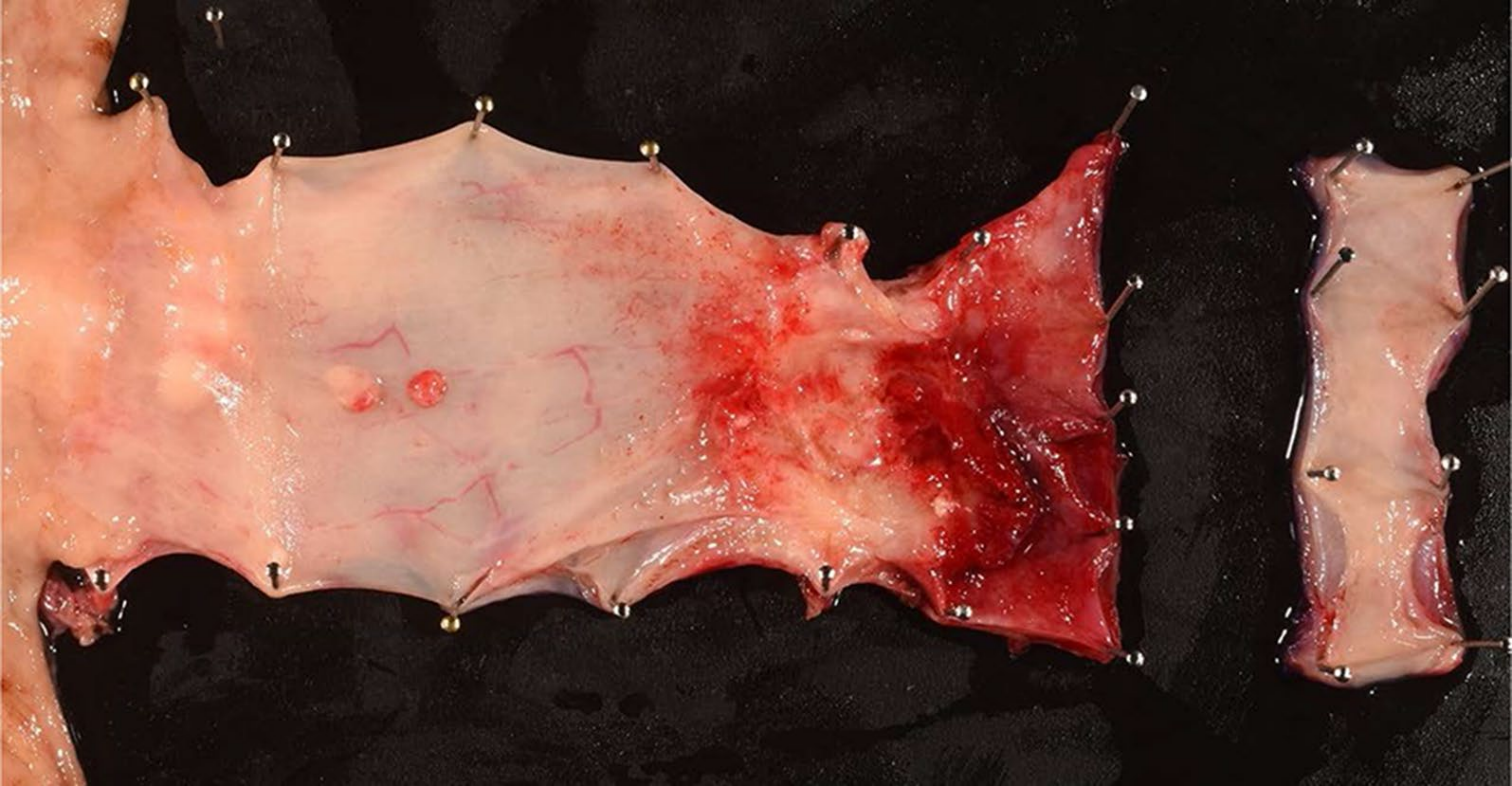
Findings of the resected specimen in Case 3

She was diagnosed as having Fanconi anemia at the age of 7 years and had undergone unrelated BMT from an unrelated HLA-mismatched male donor at the age of 15 years. She received cyclophosphamide and total body irradiation at 3 Gy as pretreatment. After the transplantation, she developed chronic GVHD manifesting as a rash and was initiated on treatment with tacrolimus and methotrexate. Fifteen years after the BMT, she became aware of difficulty in swallowing and presented to our department. She had never had an upper gastrointestinal endoscopy and was neither a habitual smoker nor drinker. Detailed examination led to the diagnosis of esophageal carcinoma (MtUt, Type 2, T3N1M0, Stage III) (Fig. 5), and like in Cases 1 and 2, she did not receive NAC, but underwent thoracoscopic thoracic esophagectomy with retrosternal gastric conduit reconstruction. She developed aspiration pneumonia postoperatively, but improved after 2 weeks of antibiotics and was discharged on postoperative day 37. The histopathological diagnosis of the resected specimen was moderately differentiated squamous cell carcinoma, Type 2, pT3N2 (5/41 metastasis positive lymph nodes) M0, pStage III, INFb, ly1, v1 (Fig. 6). She developed multiple bone metastases 6 months after the surgery and died of sepsis 7 months after the surgery.

## Discussion

SSTs commonly occur at sites with squamous epithelium, such as the skin and oral cavity, and are characterized by early onset and increasing risk of development with time after BMT [5]. Reported risk factors for SSTs include the use of immunosuppressive drugs, total body irradiation, and association with chronic GVHD, and in particular, esophageal carcinoma has been reported to be correlated with the development of chronic GVHD and long-term use of immunosuppressive drugs [5–7]. The esophageal carcinoma in all the 3 patients reported herein had the characteristics of SSTs, including early onset, total body irradiation, association with chronic GVHD, and history of use of immunosuppressive drugs (Table 1).

**Table 1:** Characteristics of the 3 cases

	Sex	Age when EC was diagnosed	pStage of EC	Hematological disorders	Donor	Immunosuppressants	Radiation	Chronic GVHD	Period from BMT to EC diagnosed
Case 1	Female	40	T3N3M0, Stage III	Acute myeloid leukemia	HLA-matched sister	Cyclophosphamide, cyclosporine	12 Gy	Pulmonary complications	9 years
Case 2	Male	45	T3N2M0, Stage III	Acute lymphocytic leukemia	HLA-matched sister	Cyclophosphamide, VP-16	13.2 Gy	Liver dysfunction	15 years
Case 3	Female	30	T3N2M0, Stage III	Fanconi anemia	Unrelated HLA-mismatched male	Cyclophosphamide, tacrolimus, methotrexate	3 Gy	Rash	15 years

EC, esophageal carcinoma; GVHD, graft-versus-host disease; BMT, bone marrow transplantation; HLA, human leukocyte antigen

A study of the late complications after BMT in 17,545 subjects enrolled in the Nationwide Survey of the Japan Society for Hematopoietic Cell Transplantation between 1990 and 2007 reported the standard incidence ratio (SIR) of SSTs by site at various periods after transplantation [7]. They reported an increase in the risk of SSTs with time after BMT and a higher risk of development of SSTs at sites of squamous epithelium, such as the oral cavity, skin and esophagus. The SIR of SSTs in the esophagus was 6.5 between 1 and 4 years, 12.6 between 5 and 9 years, and 16.8 at ≥ 10 years after BMT, indicating an increase in the risk with time after BMT and a higher risk of development in the esophagus than in other organs. Three patients reported herein had advanced cancer at the time of diagnosis, and none of the 3 patients had undergone regular screening. Thus, it is considered necessary to regularly screen patients who have undergone BMT, bearing in mind the high risk of development of esophageal carcinoma [8, 9].

Although there have been few reports until now on the treatment of SSTs, SSTs are often treated like general primary cancers [10]. No consensus has been reached on the specific aspects of treatment of esophageal carcinoma developing after BMT; however, in previously reported cases, local treatment such as endoscopic treatment, surgery or radiotherapy had been used according to the disease stage and site [3, 4, 10]. The three cases reported herein (they were 3 out of a total of 738 patients who underwent esophagectomy at our hospital between 2010 and 2020 [0.4%]) had cStage II or III advanced esophageal carcinoma, and would have been considered for NAC. Although there is no established evidence of chemotherapy for esophageal carcinoma after BMT, chemotherapy has been reported to cause serious myelosuppression; therefore, use of chemotherapy should be considered carefully [11]. We performed surgery without NAC, considering the potential adverse effects of chemotherapy on the hematopoietic function. On the other hand, all the 3 patients showed recurrence within 1 year after the surgery, and perhaps adjuvant therapy should be considered.

In regard to the postoperative management of SSTs after esophagectomy, there is a report that recommends prophylactic antimicrobial drug administration, because of the high incidence of pneumonia and the risk of severe disease [11]. All three patients herein were followed up carefully for complications of bacterial infections such as pneumonia, but only one patient developed aspiration pneumonia and improved with prompt initiation of antimicrobial therapy. In the postoperative management of SSTs patients undergoing esophagectomy, there is need to pay attention to the risk of development and severe disease of postoperative pneumonia.

The prognosis of esophageal carcinoma as an SST has been investigated using data from hematopoietic cell transplantation registries and population-based cancer registries in Japan, and there was no statistical difference in the 3-year overall survival between patients who developed esophageal carcinoma after BMT and those with primary esophageal carcinoma in the general population (after transplantation vs. primary: 34% vs. 42%, *P* = 0.40) [12]. On the other hand, three patients in our study with advanced cancer who developed recurrence early after the surgery died. As described above, we think that early detection by screening is important to improve the prognosis.

In regard to follow-up after surgery for SSTs, since there have been some reports of double cancers as SSTs, follow-up should be performed with attention paid not only to recurrence of esophageal carcinoma, but also to the occurrence of cancers of other organs, in particular, cancers arising from squamous epithelium, such as of the oral cavity, pharynx and skin [8, 9]. Endoscopists should be aware of the development of cancer of the tongue, pharynx and esophagus and should examine them carefully for early detection.

## Conclusions

Long-term follow-up is required in patients after BMT, considering the possible development of SSTs, and in regard to SSTs of the gastrointestinal tract, it should be borne in mind that the risk of esophageal carcinoma, in particular, is high.

## Data Availability

Data sharing is not applicable to this article, as no datasets were generated or analyzed during the current study.

## Abbreviations

BMT: Bone marrow transplantation
SSTs: Secondary solid tumors
HLA: Human leukocyte antigen
GVHD: Graft-versus-host disease
NAC: Neoadjuvant chemotherapy
SIR: Standard incidence ratio

## Declarations

### Authors' information

YN and MY are Senior lecturers of Department of Gastroenterological Surgery. SO is a Professor and Chairman of Department of Gastroenterological Surgery. KK is an Associate Professor of Department of Gastroenterological Surgery. TH, KY and KT are Assistant professors of Tokai University Graduate School of Medicine.
